# Body weight course in the DIAbetes and LifEstyle Cohort Twente (DIALECT-1)—A 20-year observational study

**DOI:** 10.1371/journal.pone.0218400

**Published:** 2019-06-19

**Authors:** Christina M. Gant, Ijmke Mensink, S. Heleen Binnenmars, Job A. M. van der Palen, Stephan J. L. Bakker, Gerjan Navis, Gozewijn D. Laverman

**Affiliations:** 1 Department of Internal Medicine, Meander Medical Center, Amersfoort, The Netherlands; 2 Division of Nephrology, Department of Internal Medicine, University of Groningen, University Medical Center Groningen, Groningen, The Netherlands; 3 Department of Internal Medicine/Nephrology, ZGT Hospital, Almelo and Hengelo, The Netherlands; 4 Faculty of Behavioral, Management and Social Sciences, University of Twente, Enschede, The Netherlands and Medical School Twente, Medisch spectrum Twente, Enschede, The Netherlands; Dasman Diabetes Institute, KUWAIT

## Abstract

**Background:**

Although weight gain increases risk of type 2 diabetes, real-life data on the weight course in patients with established type 2 diabetes are scarce. We assessed weight course in a real-life diabetes secondary care setting and analyzed its association with patient characteristics, lifestyle habits and initiation of insulin, glucagon like peptide-1 receptor agonists (GLP-1 RA) and sodium-glucose co-transporter-2 inhibitors (SGLT-2i).

**Methods:**

Data on weight, insulin, GLP-1 RA and SGLT-2i use were collected retrospectively (12 years) and prospectively (8 years) from patients included in the DIAbetes and LifEstyle Cohort Twente-1 (DIALECT-1, n = 450, age 63 ± 9 years, 58% men, diabetes duration [7–18] years). Lifestyle habits were assessed using validated questionnaires. The association of clinical parameters with body mass index (BMI) course was determined using linear mixed models. Patients who underwent bariatric surgery (n = 19) had a distinct BMI course and were excluded from the study.

**Results:**

Baseline BMI was 31.3 (0.3) and was higher in women, patients aged <60 years and patients with unfavorable lifestyle habits. BMI increased to 32.5 (0.3) after 12 years (P<0.001), and thereafter decreased to 31.5 (0.3) after 20 years, resulting in a similar BMI as the baseline BMI (P = 0.96, compared to baseline). Clinical parameters or initiation of insulin or SGLT-2i were not associated with BMI course. Patients who initiated GLP-1 RA declined in BMI compared to non-users (P_interaction_ = 0.003).

**Conclusions:**

High BMI that real-life patients with type 2 diabetes gained earlier in life, remained stable in the following decades. Weight loss interventions should remain a priority, and GLP-1 RA might be considered to support weight loss.

## Introduction

Adequate weight management is of utmost importance in patients with type 2 diabetes. Weight loss can remit hyperglycemia and significantly reduce risk of future micro- and macrovascular complications, even in patients with more advanced type 2 diabetes[1–3]. However, weight management in type 2 diabetes is complicated by the fact that commonly used pharmacological interventions, i.e. sulfonylureas and insulin treatment, are associated with weight gain[4,5]. On the other hand, newer blood glucose lowering pharmacological agents, i.e. glucagon-like peptide-1 receptor agonists (GLP-1 RA) and sodium-glucose co-transporter-2 inhibitors (SGLT-2i), have been associated with weight loss[4,6].

Despite the pivotal role of weight management in the course of type 2 diabetes, no long-term observational data are available on the weight course, and clinical parameters associated with weight change, of patients with established type 2 diabetes in a real life setting. Additionally, although randomized controlled trials demonstrate that insulin use is associated with weight gain, and that GLP-1 RA and SGLT-2i are associated with weight loss, observational data on the association of these agents with weight course mostly have a short, i.e. 1-year, follow up period[7–10].

Therefore, in this study we investigated body mass index (BMI) course over an 20-year period in patients included in the DIAbetes and LifEstyle Cohort Twente-1 (DIALECT-1). In addition, we analysed for possible associations of BMI course with patient characteristics, lifestyle habits, and initiation of insulin, GLP-1 RA, and SGLT-2i therapy. As we found that patients who underwent bariatric surgery during follow-up (n = 19) had a very distinct BMI course in comparison to patients who did not undergo surgery, we excluded these patients from further analyses.

## Methods

This was a longitudinal study performed in the DIAbetes and LifEstyle Cohort Twente-1 (DIALECT-1). The study population and study procedures of DIALECT-1 have been described in detail previously[11,12]. The study has been approved by local institutional review boards (Medisch ethische toetsingscommissie, METC, Twente, NL57219.044.16; METC-Groningen, 1009.68020), is registered in the Netherlands Trial Register (NTR trial code 5855), and is performed according to the guidelines of good clinical practice and the declaration of Helsinki. All participants signed an informed consent form prior to participation.

### Setting

Between September 2009 and January 2016, 450 type 2 diabetes patients were included in DIALECT-1, the flowchart of inclusion was previously described (S1 Fig) [11]. DIALECT-1 was performed in the outpatient clinic of internal medicine of the Ziekenhuis Groep Twente (ZGT hospital), located in Almelo and Hengelo, the Netherlands. The ZGT hospital is a secondary care center for diabetes treatment. In the Netherlands, referral criteria from primary to secondary health care are: inability to achieve adequate glycemic control with oral antidiabetic drugs or a standard insulin regimen, macroalbuminuria and/or estimated glomerular filtration rate (eGFR) below 60 ml/min, or multiple cardiovascular complications.

### Participants

All patients aged 18+ years visiting the internal medicine outpatient clinic for type 2 diabetes mellitus treatment were eligible for the study. Exclusion criteria were inability to understand the informed consent procedure, insufficient command of the Dutch language, or dialysis dependency. Eligible patients were selected from the electronic patient file and contacted by phone.

### Variables

At the clinic, sociodemographic characteristics, medical history, and current medications were recorded and anthropometric dimensions were measured using standard procedures. Medical history and glucose lowering drug use were additionally reviewed in the hospital electronic patient files by three different physician researchers. Smoking was assessed through a questionnaire, current smoking was defined as ≥1 cigarette/day. Physical activity was assessed using the Short QUestionnaire to ASses Health enhancing physical activity (SQUASH) questionnaire, which was previously validated[13]. Diet was assessed using a semi-quantitative validated food-frequency questionnaire (FFQ) inquiring about intake of 177 items during the last month, taking seasonal variations into account[14]. The FFQ was developed and validated at the Wageningen University and has been updated several times[14,15]. For each item, the frequency was recorded in times per day, week, or month. The number of servings was expressed in natural units (e.g., slice of bread or apple) or household measures (e.g., cup or spoon). Both questionnaires were self-administered and filled in at home. The filled-in questionnaires were checked for completeness by a trained researcher, and inconsistent answers were verified with the patients. Dietary data were converted into daily nutrient intake of macronutrients (i.e. carbohydrates, protein, fat) using the Dutch Food Composition Table of 2013[16]. Alcohol was assessed from the FFQ in units/week, where one unit corresponds with 8 grams of pure alcohol. Blood was drawn from venipuncture, for measurement of variables relevant for diabetes.

### Main outcome measure

Weight data were extracted from electronic patients file using a query for all hospital-wide weight measurements ever registered up until November 2017. Weight measurements during the follow time were performed during standard care. In the ZGT hospital, electronic scales are used for measurements which are calibrated by the technical service periodically, as recommended by the manufacturer of the devices. As the first measurements were registered in the year 2000, weight data of 18 years were available in this study. As baseline visits were performed between 2009 and 2016, retrospective weight data (weights registered before the baseline visit) varied from 9 to 16 years, and prospective weight data (weights registered after the baseline visit) varied between 8 and 1 years, respectively. Weight data were checked for completeness and errors by reviewing the electronic patient file. To account for the large interindividual variability in frequency of weight measurements, we averaged weight per three-year intervals. First, per individual patient we averaged all weight measurements performed in the same month. Then, using the average monthly weights, we averaged yearly weight. As a final step we averaged the weights of three consecutive years. Consequently, the following time intervals were defined: minus 12 through minus 10 years; minus 9 through minus 7 years; minus 6 through minus 4 years; minus 3 through minus 1 years; 0 through 2 years; 3 years through 5 years; 6 years through 8 years. Height was measured at the baseline visit (0 years), and was assumed to be stable during the follow-up period, as all patients were adults. The body-mass index (BMI) is the weight in kilograms (kg) divided by the square of height in meters (m^2^).

### Dutch Healthy Diet-index

The Dutch Healthy Diet-index score (DHD), was calculated using dietary data derived from the food frequency questionnaire, except for intake of sodium, which was derived from 24h urinary sodium excretion. An overview of the score and its components is shown in S1 Table. As data on whether consumed grains were wholegrain or refined, and data on whether consumed coffee was filtered or unfiltered were not available from the FFQ used in our study, these components in the scores were not analyzed here. Because the FFQ does not distinguish between salted and unsalted nuts, and type of tea, total nut intake and total tea intake, respectively, were used in our calculations.

The DHD score indicates the degree of adherence to the Dutch dietary guidelines published by the Health Council of the Netherlands in 2015 (see also S1 Table)[17]. The DHD score was calculated as described previously[18]. For each component, the participants receive a score on a continuous scale of 0–10, where 10 is complete adherence to the recommendation and 0 is no adherence to the recommendation.

### Statistical analysis

All statistical analyses were performed by using the Statistical Package for the Social Sciences (SPSS), version 23.0. Distributions of continuous variables were assessed by visual inspection of histograms. Normally distributed variables were presented as the mean and standard deviation. Not normally distributed variables were presented as the median and 25^th^ and 75^th^ percentile. Dichotomous variables were presented in number and percentage.

Estimated mean BMI of each 3-year time interval was determined using linear mixed model analysis, which allows for extrapolation of missing BMI data. BMI was used as the dependent factor, and time (using 3-year intervals) was a fixed factor. For all mixed model analyses, the best covariance structure was determined using Akaike’s information criterion. To study associations between clinical parameters and BMI course, we performed linear mixed models analyses with BMI as the dependent factor, and time, the clinical parameter, and their interaction (time x clinical parameter), as fixed factors. Here, P_interaction_ illustrates whether the clinical parameter significantly affects BMI course over time. To investigate the association between initiation of insulin, GLP-1 RA and SGLT-2 inhibitor use, we determined for each period whether the patient was on current treatment or not. Subsequently, we investigated the association between current drug use and BMI course by adding time, current use, and current use x time as fixed factors in the linear mixed model analyses.

## Results

Baseline characteristics of DIALECT-1 are shown in Table 1. The population consists of a secondary care type 2 diabetes population with a mean age 63 ± 9 years, median type 2 diabetes duration of 11 [7–18] years, and macro- and microvascular complications in 36% and 67% of the population, respectively. Mean BMI at baseline was 32.9 ± 6.2 kg/m^2^, illustrating that this was a predominantly obese population, with 30% of patients being overweight, and 65% being obese.

**Table 1:** Baseline, nutritional, and pharmacological characteristics of type 2 diabetes patients in DIALECT-1.

		*Number of patients*	
Men, n (%)		*450*	261 (58)
Age, years		*450*	63 ± 9
Years since type 2 diabetes diagnosis, years		*450*	11 [7–18]
	≥ 15 years, n (%)	*450*	157 (35)
Weight, kg		*450*	98 ± 19
Length, cm		*450*	172 ± 9
Body mass index, kg/m2		*450*	32.9 ± 6.2
	<25, n (%)		23 (5)
	25–30, n (%)		134 (30)
	>30, n (%)		291 (65)
Bariatric surgery during follow up, n (%)		*450*	19 (4)
Macrovascular disease, n (%)		*450*	161 (36)
Microvascular disease, n (%)		*446*	295 (67)
	Chronic kidney disease, n (%)	*446*	188 (42)
Blood pressure, mmHg		*448*	139/76 ± 16/10
Heart frequency (beats/min)		*448*	74 ± 13
HbA1c, mmol/mol		*448*	57 ± 12
LDL cholesterol, mmol/l		*428*	2.0 ± 0.8
*Lifestyle factors*			
Adherence to guideline physical activity		*433*	253 (56)
Smoking		*450*	
	Current		75 (17)
	Former		238 (53)
Alcohol intake		*450*	
	None, n (%)		155 (34)
	1–13, n (%)		208 (46)
	14+, n (%)		61 (14)
Total Dutch Healthy Diet index score		*433*	74 ± 13
*Pharmacological treatment*			
Metformin, n (%)		*450*	333 (74)
Sulfonylureas, n (%)		*450*	114 (25)
DPP4-inhibitors, n (%)		*450*	19 (4)
Pioglitazine, n (%)		*450*	7 (2)
GLP-1 receptor antagonists, n (%)		*450*	45 (10)
SGLT-2 inhibitors, n (%)		*450*	4 (1)
Insulin use, n (%)		*450*	297 (69)

DPP4, dipeptidylpeptidase 4; GLP, glucagon-like peptide; SGLT, sodium-glucose co-transporter.

### Body mass index time course

**Fig 1:**
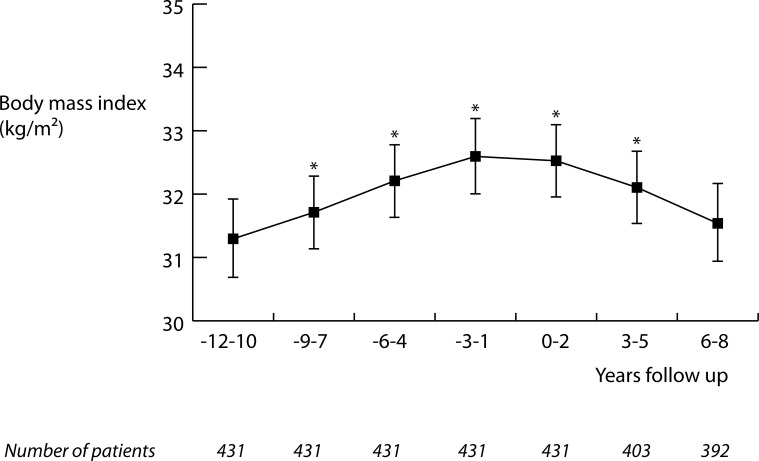
Body mass index course using estimated means in patient with type 2 diabetes included in DIALECT-1, during a retrospective follow-up period of 12 years and a prospective follow-up period of 8 years. Estimated means were calculated using linear mixed model analysis. The brackets represent 95% confidence interval of the mean. * Significant estimated mean change from starting BMI (P<0.05).

In Fig 1, BMI course during the follow-up period is illustrated. During follow-up 39 patients deceased, and these patients were excluded in estimated mean calculations of the corresponding time periods. Mean BMI in the starting time interval (-12 to -10 years) was 31.3 (0.3) kg/m^2^, see also Table 2. During the first 12 to 14 years we observed a steady and significant rise in BMI towards a mean BMI of 32.5 (0.3) kg/m2 in the 0–2 year time interval (P<0.001, Fig 1, Table 2). However, after this initial rise, BMI again decreased to the starting BMI with a mean of 31.5 (0.3) in the final time interval (6–8 years; P = 0.96 versus starting BMI). Therefore, although there was a significant change in BMI during the follow up period (P_interaction_<0.001), in the total 18 to 20 year follow-up period we did not observe significant BMI change in this real life type 2 diabetes population.

**Table 2:** Patient characteristics associated with body mass index time course over an 12-year retrospective, and 8 year prospective follow period, in patients included in DIALECT-1.

			BMI at starting interval* (-12 to -10 years)		ΔBMI to baseline time interval (0 to 2 years)		ΔBMI to final time interval (6 to 8 years)		BMI trend
		No. of patients	BMI (SE)	P-value BMI difference between subgroups	ΔBMI (SE)	P-value ΔBMI difference between subgroups	ΔBMI (SE)	P-value ΔBMI difference between subgroups	P_interaction_
All patients		431	31.3 (0.3)		1.2 (0.3)	<0.001	0.3 (0.3)	0.95	
Gender									0.10
	Men	252	30.4 (0.4)	<0.001	1.0 (0.3)	0.001	0.4 (0.3)	0.78	
	Women	179	32.6 (0.6)		1.3 (0.3)	<0.001	0.2 (0.3)	0.97	
Age									0.44
	<60 years	121	32.6 (0.6)	<0.001	0.8 (0.4)	0.03	-0.2 (0.7)	>0.99	
	60-70 years	225	31.9 (0.4)		0.9 (0.3)	0.02	0.2 (0.3)	>0.99	
	> 70 years	85	29.4 (0.7)		1.3 (0.7)	0.30	-0.1 (0.8)	>0.99	
Years since diabetes diagnosis									0.56
	0-15 years	300	31.3 (0.4)	0.89	1.3 (0.4)	0.005	0.2 (0.4)	0.99	
	> 15 years	131	31.4 (0.5)		1.0 (0.3)	0.01	0.3 (0.4)	0.98	
Smoking									0.49
	Never smoked	132	31.9 (0.5)	0.43	1.0 (0.4)	0.05	-0.1 (0.5)	>0.99	
	Former smoker	229	30.9 (0.4)		1.4 (0.4)	0.001	0.4 (0.4)	0.93	
	Current smoker	70	31.6 (0.8)		0.9 (0.7)	0.81	0.1 (0.8)	>0.99	
Alcohol intake									0.34
	None	145	32.7 (9.5)	0.01	0.9 (0.6)	0.59	-0.5 (0.6)	0.95	
	1-13 units/week	202	30.5 (0.5)		1.3 (0.3)	<0.001	0.5 (0.3)	0.59	
	14+ units/week	58	31.0 (0.9)		0.8 (0.5)	0.47	-0.6 (0.3)	0.15	
Dutch Healthy Diet score									0.82
	Genderspecific lowest tertile	138	32.5 (0.6)	0.005	1.4 (0.4)	0.01	0.3 (0.5)	>0.99	
	Genderspecific middle tertile	140	31.4 (0.5)		1.4 (0.5)	0.05	0.0 (0.5)	>0.99	
	Genderspecific highest tertile	138	29.9 (0.5)		1.3 (0.4)	0.003	0.9 (0.4)	0.17	
Adherence to the guideline physical activity									0.04
	Not adherent	173	32.7 (0.5)	<0.001	1.4 (0.5)	0.05	-0.2 (0.5)	>0.99	
	Adherent	242	30.4 (0.4)		1.2 (0.3)	<0.001	0.7 (0.3)	0.11	
Macrovascular disease at baseline									0.78
	No macrovascular disease	274	31.4 (0.4)	0.76	1.3 (0.4)	0.004	0.4 (0.4)	0.88	
	Macrovascular disease	157	31.1 (0.5)		1.0 (0.3)	0.02	0.0 (0.4)	>0.99	
Chronic kidney disease at baseline									0.48
	No CKD	242	31.2 (0.4)	0.75	1.2 (0.4)	0.01	0.4 (0.4)	0.96	
	CKD	186	31.3 (0.5)		1.3 (0.3)	0.001	0.2 (0.4)	>0.99	
HbA1c on target (<53 mmol/mol)									0.53
	Not on target	282	31.6 (0.4)	0.72	0.9 (0.3)	0.004	-0.1 (0.3)	>0.99	
	On Target	149	30.8 (0.6)		1.7 (0.5)	0.01	0.7 (0.5)	0.66	
*Pharmacological agents*									
Metformin									0.06
	No use	108	29.6 (0.6)	<0.001	2.7 (0.6)	<0.001	1.9 (0.5)	0.006	
	Use at baseline	323	31.8 (0.3)		0.7 (0.3)	0.03	-0.3 (0.3)	0.95	
Sulfonylureas									0.11
	No use	322	30.9 (0.3)	0.02	1.3 (0.3)	<0.001	0.5 (0.3)	0.55	
	Use at baseline	109	32.5 (0.7)		1.6 (0.7)	<0.001	0.0 (0.6)		

* The follow-up period was divided in 3-year time intervals.

BMI, body mass index; SE, standard error of the mean; CKD, chronic kidney disease

### Parameters associated with body mass index

Consequently, we investigated which clinical characteristics were associated with differences in BMI (Table 2). Factors associated with a higher mean BMI in the first time interval (-12 to -10 years) were: female gender; lower age; lower alcohol intake; lower DHD-score; non-adherence to the guideline on physical activity; metformin use at baseline; and sulfonylurea use at baseline.

### Time course of body mass index and its association with clinical parameters

In most subgroups, there was an initial rise in BMI from the -12 to -10 years period to the 0 to 2 years period, and a consequent fall in BMI to the 6 to 8 years period, resulting in a similar BMI as in the starting period. Only in patients not on metformin treatment during the baseline visit, BMI was significantly higher at the end of the follow-up period, BMI change +1.9 (0.5) kg/m^2^ (P = 0.006). BMI course in the total follow-up period was not significantly different for all parameters, except for adherence to the guideline on physical activity (P_interaction_ = 0.04).

**Fig 2:**
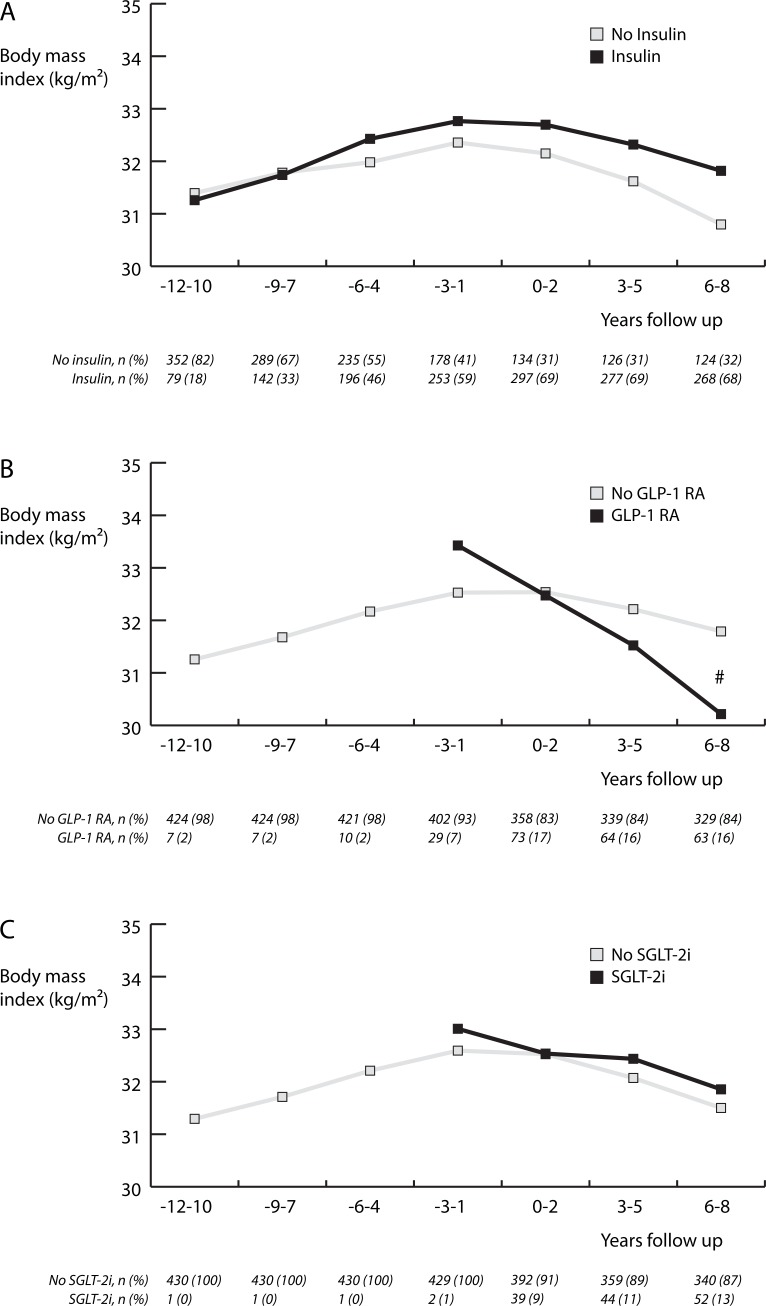
Weight course using estimated means by a break-up of A) insulin, B) GLP-1 RA and C) SGLT-2i use in patient with type 2 diabetes included in DIALECT-1. Estimated means were calculated using linear mixed model analyses. BMI course in patients who started on GLP-1 RA was significantly different from those who did not (P_interaction_ = 0.003). There was no difference in overall BMI course between the insulin groups (P_interaction_ = 0.46) and SGLT-2i groups (P_interaction_ = 0.83). # Significant difference in estimated mean BMI between the groups. GLP-1 RA, glucagon-like peptide-1 receptor agonists; SGLT-2i, sodium-glucose-cotransporter-2 inhibitors.

In Fig 2 we illustrate the association between different pharmacological interventions on BMI course over the years. In the starting time interval of -12 to -10 years, 79 (18%) patients used insulin, 7 (2%) patients used GLP-1 RA and 1 (0%) patient used a SGLT-2i. This increased to 268 (68%), 63 (16%) and 52 (13%) patients, respectively, in the final time interval of 6 to 8 years. Starting BMI was equal in those not using and using insulin (31.4 (0.3) vs 31.3 (0.4) kg/m^2^, P = 0.88). However, those not using insulin had a lower ending BMI, although not statistically significant (30.8 (0.5) vs 31.8 (0.3) kg/m^2^, P = 0.08). There was no significant difference in BMI course in non-insulin users and insulin users (P_interaction_ = 0.46). Patients who started on GLP-1 RA had a significantly different BMI course than those who did not (P_interaction_ = 0.003), illustrated by a non-significant higher BMI in the first time interval and a significantly lower end BMI in GLP-1 RA users (30.2 (0.5) vs 31.8 (0.3) kg/m^2^ in non-users, P = 0.04). SGLT-2i use was not associated with a different BMI course in comparison to no use (P_interaction_ = 0.83), and there were no differences in BMI between SGTL-2i users and non-users on different time points. Addition of age, gender, metformin use, or adherence to the guideline on physical activity did not significantly change the models.

## Discussion

In this study we investigated BMI course in a cohort of patients with type 2 diabetes, in a real-life secondary care treatment setting. We found that in a follow-up period of 18 to 20 years there was no change in end BMI compared to the starting BMI. Furthermore, there were no associations between patient characteristics, lifestyle habits or initiation of insulin or SGLT-2i therapy and BMI course over the years. However, patients who were started on glucagon-like peptide-1 receptor agonists had a greater BMI decline than those who did not initiate GLP-1 RA therapy, resulting in a lower BMI at the end of the follow-up period. To our knowledge, this is the first report on long-term term BMI course in patients with advanced type 2 diabetes, and its association with pharmacological agents.

Our finding that in patients with type 2 diabetes over a 18 to 20 year observational period BMI first increased, and thereafter decreased to starting BMI, is in contrast to the general belief that in diabetes patients, weight gain is steady throughout the years. Previously, in an observational study in patients with type 2 diabetes for ≥5 years, Chaudry et al. reported a weight gain of 0.2 ± 0.2 kg/year, with a mean follow-up duration of 9 years[19]. In the general Dutch population, in a similar follow-up period as in this study, in the same age category, a total weight gain of 5.6 kg in men and 1.8 kg in women was reported[20]. However, compared to the general Dutch population, starting BMI in this diabetes population was considerably higher (25.5 vs 31.3 kg/m^2^ in DIALECT-1). Why our findings differ from earlier reports in type 2 diabetes and the general Dutch population is unknown. Previous research demonstrated a clear association between weight gain early in life and subsequent increased risk of developing type 2 diabetes[21,22]. Perhaps in this population, weight gain occurred during adolescence or early to middle adulthood until a BMI plateau was reached, leading to the stable obesity seen in this study. This is supported by a long-term observational study by Looker et al., which demonstrated a BMI increase in the five years before the diagnosis of type 2 diabetes, and stable weight, and in some cases even weight loss, in the years following the diagnosis[23]. Alternatively, perhaps our findings could demonstrate effective lifestyle counseling in our setting of routine clinical care. However, this is in contrast to our previous report that lifestyle habits in DIALECT-1 are not different from those of the general population[12]. Another possible explanation could be that although no changes in BMI are observed, changes in body composition do occur. In regard to body composition, especially fat mass contributes to insulin resistance, and a body composition with high fat mass and low muscle mass is associated with an increased risk of cardiovascular events[24,25]. Kyle et al. demonstrated that after 60 years of age, decline in fat free mass and appendicular skeletal muscle is accelerated, while fat mass continued to increase until 75 years of age[26]. As the mean age in this population is 63 ± 9 years, it is conceivable that similar changes took place in the follow-up period.

Additionally, we found that BMI course did not differ between patients who remained on only oral antidiabetic drugs during the follow-up period, or patients who used/started using insulin therapy. This was a surprising finding, as insulin therapy is commonly associated with weight gain in both intervention studies and observational studies[4,5,19,27]. In type 2 diabetes patients with a disease duration of ≥5 years, Chaudry et al. reported a mean weight gain of 0.44 ± 0.1 kg/year in insulin users vs -0.24 ± 0.09 kg/year weight loss in patients with only oral glucose lowering drugs. Our findings are supported by a previous study, by Nichols et al., which also demonstrated less than expected weight gain 1 year after initiation of different glucose lowering therapies in a real life setting[28]. Possibly in the population we studied, patients with the most pronounced weight gain after initiation on insulin might have ceased insulin use or had other interventions aimed at weight loss, such as bariatric surgery or starting on GLP-1 RA. Our findings illustrate that results from controlled clinical trials do not represent the real world of clinical practice, where many uncontrollable factors may contribute to biological processes.

Initiation of GLP-1 RA was associated with a significant decline in BMI over the years. Although patients on GLP-1 RA had a higher starting BMI than those who did not initiate GLP-1 RA therapy, BMI at the end of the follow-up period was lower in GLP-1 RA users. The higher starting BMI in GLP-1 RA users reflects the fact that in the last decade these agents were only reimbursed in patients with a BMI >35kg/m^2^. Weight loss due to GLP-1 RA was previously described in clinical trials[29,30]. Here we illustrate that also in a real-life setting, GLP-1 RA is associated with significant and clinically relevant weight loss. However, caution is warranted when interpreting these data, as this is an observational study, and therefore patient or physician related factors could have influenced the physician’s decision whether or not to initiate or continue GLP-1 RA therapy. For example, possibly patients who were initiated on GLP1-RA with a limited response on HbA1c might promptly be switched to insulin therapy instead. In contrast, we found no association between SGLT-2i use and BMI course. This could be due to the fact that only a very small number of patients in our study was started on SGLT-2i (in total 13%), and only in the last two intervals of the follow-up period.

We also studied whether lifestyle related factors were associated with BMI and BMI course. We found that patients with more favorable dietary habits, illustrated by a higher DHD-index and adherence to the guideline on physical activity, had a significantly lower BMI than patients with unhealthier lifestyle habits. A healthier lifestyle was, however, not associated with BMI course over the years. This is unsurprising, because changes in lifestyle are required to achieve weight loss, namely reducing overall dietary caloric intake and increasing dietary quality and physical activity. Our findings do support that patients should be encouraged to adhere to the guidelines on healthy lifestyle.

It should be noted that in our study, patients who underwent bariatric surgery were excluded from analyses on factors associated with weight course. This could have induced selection bias in our study. Also, caution is warranted when extrapolating our results to patients with a history, or on the waiting list for, bariatric surgery.

The most important strength of our study is the fact that our data represent type 2 diabetes patients in a real life secondary care setting. Additionally, due to the broad inclusion criteria there is minimal inclusion bias. There were also some limitations in our study. Due to missing data we used estimated means to calculate BMI per time interval instead of using measured BMI values. Also, longitudinal data on lifestyle was not available, therefore the effect of possible lifestyle interventions aimed to reduce weight was not included in the analysis. Furthermore, as our study population only consists of patients with diabetes, no direct comparison to a non-diabetes control population could be made. Lastly, weight measurements in the follow-up period were performed during routine care, and therefore were not standardized.

What should be the implications of our study? Our data illustrate that in a real-life population of patients with type 2 diabetes in secondary care BMI first increases, and then decreases to starting BMI over the years. However, in this very high risk population, with previous cardiovascular events in 36% of patients, and kidney disease in 42% of patients, weight reduction is of utmost importance to reduce insulin resistance and risk of future cardiovascular events. Previously, we demonstrated low achievement of target values in this population; the blood pressure target was reached in 53% of patients, and HbA1c only in 36%, which was paralleled by a high degree of obesity and insulin resistance[11,31]. Weight loss has been associated not only with a reduction in HbA1c, blood pressure, LDL-cholesterol, but also a reduction in the risk of long-term complications such as cardiovascular disease and diabetic nephropathy[1,32–34]. Therefore, more aggressive approaches to reduce weight are warranted.

In terms of pharmacology, our data illustrate some benefit of GLP-1 RA on weight management in type 2 diabetes patients. Additionally, GLP-1 RA have also been associated with a reduction of major adverse cardiovascular endpoints[35]. Therefore, in patients with insufficient glycemic control with oral glucose lowering drugs, starting GLP-1 RA therapy instead of insulin therapy might be considered to support weight loss management.

In conclusion, in this long-term observational study on BMI course in patients with type 2 diabetes in a real life setting, we demonstrated that in a follow period of 18–20 years BMI initially increased, and thereafter decreased to starting BMI. Additionally, initiation of insulin therapy had no significant effect on BMI course, while GLP-1 RA use was associated with a significant BMI decline. Therefore, in overweight type 2 diabetes patients, interventions aimed at weight loss should be a top priority, and GLP-1 RA could be considered as a pharmacological agent to support weight loss.

## Supporting information

S1 Fig Patient inclusion flowchart. (EPS)

S1 Table Components and scoring of the Dutch Healthy Diet-index. (DOCX)

S1 Dataset (SAV)
