# Supplementary material for: Body weight course in the DIAbetes and LifEstyle Cohort Twente (DIALECT-1)—A 20-year observational study
Source: PLoS One. 2019 Jun 19;14(6):e0218400. doi: 10.1371/journal.pone.0218400 (PMC6583961; doi:10.1371/journal.pone.0218400)
Supplement: S1 Table — (DOCX) [file pone.0218400.s002.docx]

**Supplementary Table 1**. Components and scoring of the Dutch Healthy Diet-index

| Dietary pattern and components | | Minimum score | Maximum score |
| --- | --- | --- | --- |
| Dutch Heatlhy Diet-index (DHD) | | 0 points | 10 points |
| 1 | Vegetables | 0 g/d | ≥200 g/d |
| 2 | Fruit | 0 g/d | ≥200 g/d |
| 3 | Wholegrain products^a^ | No consumption of wholegrain products | No consumption of refined products |
| 4 | Legumes | 0 g/d | ≥10 g/d |
| 5 | Nuts | 0 g/d | ≥15 g/d |
| 6 | Dairy (including milk or yoghurt) | 0 g/d or ≥750 g/d | 300-450 g/d |
| 7 | Fish | 0 g/d | ≥15 g/d |
| 8 | Black or green tea | 0 g/d | ≥450 g/d |
| 9 | Fats and oils | Ratio liquid cooking fats to solid cooking fats ≤0.6 | Ratio liquid cooking fats to solid cooking fats ≥13 |
| 10 | Coffee^a^ | Any consumption of unfiltered coffee | No consumption of unfiltered coffee |
| 11 | Red meat | ≥100 g/d | ≤45 g/d |
| 12 | Processed meat | ≥50 g/d | 0 g/d |
| 13 | Sweetened beverages and juices | ≥250 g/d | 0 g/d |
| 14 | Alcohol | ≥20 g/d (women) | ≤10 g/d (women) |
|  |  | ≥30 g/d (men) | ≤10 g/d (men) |
| 15 | Sodium | ≥3.8 g/d | ≤1.9 g/d |
|  |  |  |  |

^a^ Component was not included in calculation of the final score
